# In Vivo Evaluation of Different Collagen Scaffolds in an Achilles Tendon Defect Model

**DOI:** 10.1155/2018/6432742

**Published:** 2018-08-08

**Authors:** Carolin Gabler, Jan-Oliver Saß, Susann Gierschner, Tobias Lindner, Rainer Bader, Thomas Tischer

**Affiliations:** ^1^Rostock University Medical Center, Department of Orthopedics, Biomechanics and Implant Technology Laboratory, Rostock, Germany; ^2^Rostock University Medical Center, Core Facility Multimodal Small Animal Imaging, 18057 Rostock, Germany

## Abstract

In the present study, a newly introduced bovine cross-linked collagen scaffold (test material) was investigated in vivo in an Achilles tendon defect model and compared to a commercially available porcine collagen scaffold (control material). In total, 28 male Sprague Dawley rats (about 400 g) were examined. The defined Achilles tendon defect of 5 mm of the right hind limb was replaced by one of the scaffold materials. After euthanasia, the hind limbs were transected for testing. Biomechanical evaluation was carried out via tensile testing (n = 8 each group, observation time: 28 days). Nonoperated tendons from the bilateral side were used as a control (native tendon, n = 4). For the histological evaluation, 12 animals were sacrificed at 14 and 28 days postoperatively (n = 3 each group and time point). Stained slices (Hematoxylin & Eosin) were evaluated qualitatively in terms of presence of cells and cell migration into scaffolds as well as structure and degradation of the scaffold. All transected hind limbs were additionally analyzed using MRI before testing to verify if the tendon repair using a collagen scaffold was still intact after the observation period. The maximum failure loads of both scaffold materials (test material: 54.5 ± 16.4 N, control: 63.1 ± 19.5 N) were in the range of native tendon (76.6 ± 11.6 N, p ≥ 0.07). The stiffness of native tendons was twofold higher (p ≤ 0.01) and the tear strength was approximately fivefold higher (p ≤ 0.01) compared to the repaired tendons with both scaffolds. Histological findings indicated that neither the test nor the control material induced inflammation, but the test material underwent a slower remodeling process. An overall repair failure rate of 48% was observed via MRI. The experimental data of the newly developed test material showed similar outcomes compared to the commercially available control material. The high repair failure rate indicated that MRI is recommended as an auxiliary measurement tool to validate experimental data.

## 1. Introduction

Tendon regeneration, e.g., after rotator cuff tears, is known to be a complex and slow process, and the healing of tendon repair still remains a clinical challenge. Depending on individual factors (e.g., patient's age, tendon quality, and tear size) high rerupture rates can be observed [1–3]. Therefore, scaffold devices for tendon augmentation, whether biologic or synthetic, have been introduced to increase healing rates [4–6]. It is important to note that the absence of approval from the health authorities limits the clinical use of some graft materials in many countries. For example, in Germany, allografts are subjected to regulations based on transplantation law. In Japan, the use of allografts is also not approved and therefore the use of autografts is common [7].

The biomechanical behavior of graft materials in vitro was characterized by means of uniaxial mechanical tests [8, 9]. The results can be adjusted by varying the sample characteristics of the material tested such as thickness [10] or the processing method of the material [11], independently from graft source. Therefore, clinically relevant scaffold constructs should be performed to produce qualitative evidence. In recent years, several clinical trials have evaluated the functional outcome of the augmented rotator cuff repair (RCR) with the use of different scaffold devices. Most of these trials were quite limited as they were often retrospective case series with small patient populations, without control groups, and produced controversial results [12, 13]. Based on the recent literature, allografts made of acellular human dermis are thought to provide the most beneficial clinical outcome with low rerupture rates compared to other scaffolds [14–16]. In the current literature, the clinical outcome of xenografts is discussed controversially [7, 17, 18]. Thus, the source of the graft plays an important role [4, 12]. In particular, collagen-based grafts made from porcine small intestine submucosa (SIS) are known to end up in suboptimal results and may promote postoperative inflammatory reactions. Therefore, the use of porcine SIS xenografts (Restore, DePuy) for augmentation in RCR is not recommended [12, 19]. Ciampi et al. [20] reported that RCR using bovine pericardium-derived patches (Tutopatch, RTI Surgical, Inc. Alachua, FL, USA) showed significantly lower healing rates compared to synthetic grafts (Repol Angimesh, Angiologica BMSrl, Pavia, Italy). However, the healing rate was not significantly lower than RCR without a graft. Further clinical data showed that xenografts, based on dermal collagen, led to more promising results [5, 21–23]. However, it is not known if there are differences in clinical outcome with respect to the dermal graft source (porcine versus bovine).

Currently, there are two graft materials based on bovine dermis available for clinical use (TissueMend, Stryker Corp., Mahwah, NJ, USA, and Bio-Blanket, Kensey Nash Corp., PA, USA), but only few results have been published on clinical outcomes. Sears et al. [24] compared the clinical outcome of an allograft (GraftJacket, Wright Medical Arlington, TN, USA), a porcine dermal extracellular matrix (ECM) (Conexa, Tornier Inc., Bloomington, MN, USA), and a bovine dermal ECM (TissueMend, Stryker Corp.) in a retrospective case study. Significant differences in clinical outcome were not found between the different patches. However, the study was strongly limited by the small sample size.

The inconsistent clinical outcomes using scaffolds for tendon augmentation underline that there is still a need for new scaffold materials. Therefore, we have developed a new scaffold material, based on bovine collagen and a chemical cross-linking process. The material has been already tested in vitro and showed favorable biomechanical, biochemical, and cell biological properties [25]. In the present pilot animal study, the newly introduced bovine collagen scaffold was investigated with regard to functional outcomes and remodeling using an Achilles tendon defect model in rats.

## 2. Materials and Methods

### 2.1. Scaffolds

Two collagen scaffold materials were evaluated. As the test material, a newly developed scaffold based on bovine collagen was used, as described previously [25]. Briefly, for scaffold preparation, bovine dermal collagen was treated chemically with NaOH, H_2_O_2_, and HCl in order to remove noncollagenous proteins, fatty acids, and cells and to inactivate viruses. The purified dermal collagen was processed into a matrix with longitudinally orientated fibrils consisting mainly of collagens type I, type III, and type V. This predefined matrix structure was first stabilized by freeze-drying within a temperature range of 55 to 65°C and further by chemical cross-linking. Then, the freeze-dried matrix was subjected to an aqueous epoxide solution with a concentration of 0.19% (w/w). Before further use, the cross-linked collagen matrix was successively washed with reverse osmosis (RO) water to remove any free epoxide. For the control group, DX Reinforcement scaffolds (Arthrex, Inc., Naples, FL, USA) based on porcine dermal extracellular matrix with no cross-linking were used.

Before testing, the test material was hydrated in saline solution (NaCl, 0.9%) for at least 30 min (thickness after rehydration: 0.89 ± 0.04 mm). The control material was delivered hydrated (thickness: 1.43 ± 0.16 mm). Test samples for* in vitro* and* in vivo* evaluation were obtained from two patches of one charge for the test material and from two patches of two charges for the control material.

### 2.2. Biomechanical Testing of Scaffold Materials In Vitro

As we used samples from new charges, we repeated the initial biomechanical testing [25] according to Barber and Aziz-Jacobo [8]. Before animal testing, additionally a suture retention test was performed according to Barber and Aziz-Jacobo [8]. Samples were bisected and one vertical stitch with No. 2 FiberWire (Arthrex, Inc., Naples, FL, USA) was passed to the distal end of the scaffold with a distance of 5 mm from the tissue edge. The scaffold was mounted on the upper grip with roughened chuck jaws in the testing machine; the suture was fixed with a sample grip with corrugated chuck jaws. The start length was 3 cm and the predetermined tearing location was centered. The destructive test was conducted again with a distraction rate of 12.5 mm/s.

For all biomechanical evaluations of the test material, care was taken that the load was applied longitudinally to the orientation of collagen fibers (according to native in vivo situation). Six samples of the test material were tested. Only four samples of the control material were tested due to the limited quantity of the material. The orientation of the control material during testing was not required, since this graft has no directional fiber alignment.

### 2.3. Animal Testing

**Figure 1:**
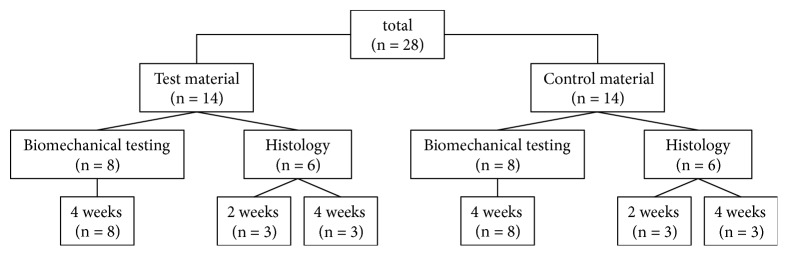
Overview of the allocation of rats and experiments used in present study.

For in vivo testing of both scaffold materials, a total of 28 male Sprague-Dawley rats (Janvier Labs, Le Genest-Saint-Isle, France) weighing 404 ± 20 g were used. The study was approved by the local animal research committee (LALLF MV, reference number 7221.3-1-036/15). Rats were kept in an animal facility, where temperature and light/dark cycle (12:12 hours) were controlled, and access to standard food and water was provided ad libitum. Animals were randomly divided into two groups: the test material group, where the Achilles tendon defect was replaced by the bovine collagen scaffold, and the control group, where the DX Reinforcement graft material was used as the scaffold. For functional and biomechanical evaluations, the postoperative observation time was 28 days (n = 8 each group). To determine the optimal number of animals, an a priori analysis was performed for the mean of two independent samples using G*∗*Power (version 3.1.9.2). For the histological evaluation, in total 12 animals were sacrificed 14 and 28 days postoperatively (n = 3 each group and each time point). The allocation of the rats is shown in Figure 1.

**Figure 2:**
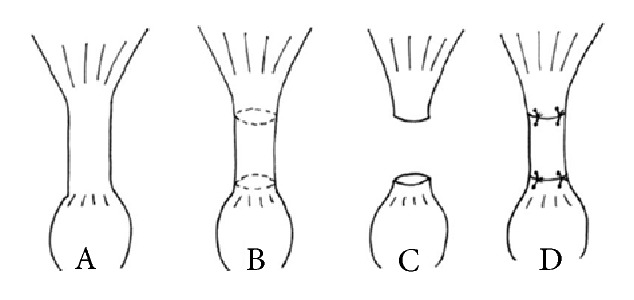
Schematic illustration of implantation procedure. A: preparation of the Achilles tendon, B: marking defined defect length of the Achilles tendon, C: setting the defined defect, D: implantation of the scaffold material, suturing with two single stitches at each end.

Surgery was performed by an experienced orthopedic surgeon (TT), who underwent additionally a training on cadaveric rats before. For surgery, the animals were anesthetized with medetomidine (150 *μ*g/kg), midazolam (2 mg/kg), and fentanyl (5 *μ*g/kg) due to an intraperitoneal injection. The right Achilles tendon was dissected and freed from soft tissue and the M. plantaris tendon was removed. Each rat underwent a transection of 5 mm of the Achilles tendon. Due to differences of sizes of the animals, the transection was set individually in the middle part of the tendon. A 5 mm scaffold of either the new bovine material (n = 14) or the porcine control material (n = 14) was replaced in the defect and the remaining ends were refixed with two single stitches (Vicryl® 4-0, Ethicon, Somerville, NJ, USA) (Figure 2). Collagen fibers of the test material scaffold device were aligned longitudinally to the orientation of the Achilles tendon.

Finally, anesthesia was antagonized by a subcutaneous (s.c.) dose of atipamezole (750 *μ*g/kg), flumazenil (200 *μ*g/kg), and naloxone (120 *μ*g/kg). Postoperative analgesia was provided through an intramuscular injection of 0.5 ml metamizol (0.5 g/ml) immediately after surgery, as well as orally via the drinking water (30 drops 500 mg/ml metamizol per 0.5 l) for three days. Animals were allowed to move freely in their cages after surgery. In the first postoperative week, they were kept individually per cage to prevent adverse events like fighting or mutually gnawing on surgical sites. At the end of the observation period, the animals were euthanized by an intracardiac injection with an overdose of pentobarbital sodium (80 mg/kg) under anesthesia.

### 2.4. Imaging Analysis

After scarification of the animals, hind limbs were transected proximal to the knee joint, and magnetic resonance imaging (MRI) scans were acquired using 7.0 Tesla scanner (BioSpec 70/30, Bruker, Ettlingen, Germany). T_2_-weighted TurboRARE sequences in axial, coronal, and sagittal plane were recorded (TE/TR: 28/4400 ms, spatial in-plane resolution: 0.12 mm, slice thickness: 0.7 mm, matrix size (sagittal/frontal/transversal): 338 × 304 / 320 × 166 / 280 × 196, FoV (sagittal/frontal/transversal): 40.5 mm x 36.5 mm / 38.3 mm × 20 mm / 33.5 mm x 23.5 mm, RARE factor: 8, averages: 5). Afterwards, specimens for biomechanical testing were wrapped in gauze soaked with NaCl solution (0.9%) and stored at −20°C. Specimens for histomorphometric analyses were fixed in buffered formalin (4%).

**Figure 3:**
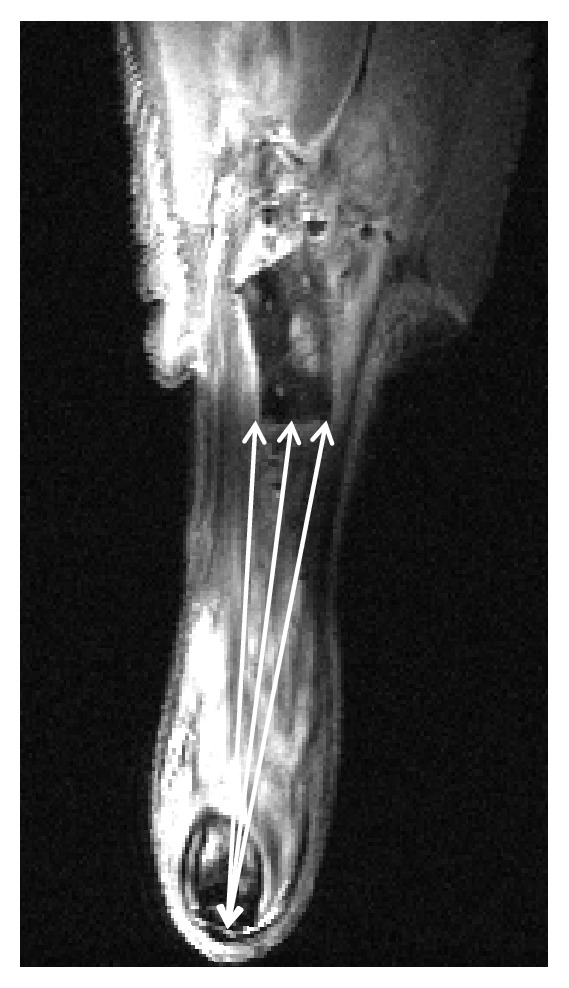
T_2_-weighted MR image of an experimental hind limb repaired with a collagen scaffold (dorsal view). Arrows indicate measurement of the distance between the distal edge of the scaffold and tendon insertion into the calcaneus.

For analysis, the software Amira 5.4.1 (Thermo Fisher Scientific, USA) was used. The distance between the distal edge of the scaffold and the tendon insertion on the calcaneus was measured. Three measurements per view were executed, and mean value was calculated for each sample, as shown in Figure 3. Additionally, the junction between the scaffold and the native tendon was evaluated. It was determined whether the reconstruction was still intact or if it had failed during the observation time. Failure was defined if either the position of the scaffold was obviously slipped into the proximal direction (distance to calcaneus > 10 mm) and/or the musculotendinous junction was elongated.

### 2.5. Functional Evaluation

A gait analysis was performed for the animals planned for biomechanical testing. A modified method for the Achilles Functional Index (AFI) described by Murrel et al. [26], according to Kurtz et al. [27], was used. Therefore, the hind paws of the rats were colored by dipping a sponge soaked with nontoxic food dye. Animals were then allowed to walk a confined walkway prepared with white paper on the floor of the corridor, leaving paw prints on the paper. Gait was recorded preoperatively, at day 4 and day 7, and then at weekly intervals up to day 28. The papers were scanned and measurements of from paw prints were performed with GIMP 2.8.20 (GIMP, the GIMP Team). Measurements included print length (PL), total spreading (TS, distance between first and fifth toes), and intermediary spreading (IT, distance between second and fourth toes). Three left and three right paw prints were evaluated and averaged each time point to calculate the corresponding factors (PLF, TSF, and ITF) according to [21]. Murrel's formula was used for determination of AFI:

(1) $$\begin{array}{c} AFI=74\left(\;PLF\;\right)+161\left(\;TSF\;\right)+48\left(\;ITF\;\right)-5 \end{array}$$

[26].

### 2.6. Biomechanical Testing In Vivo

**Figure 4:**
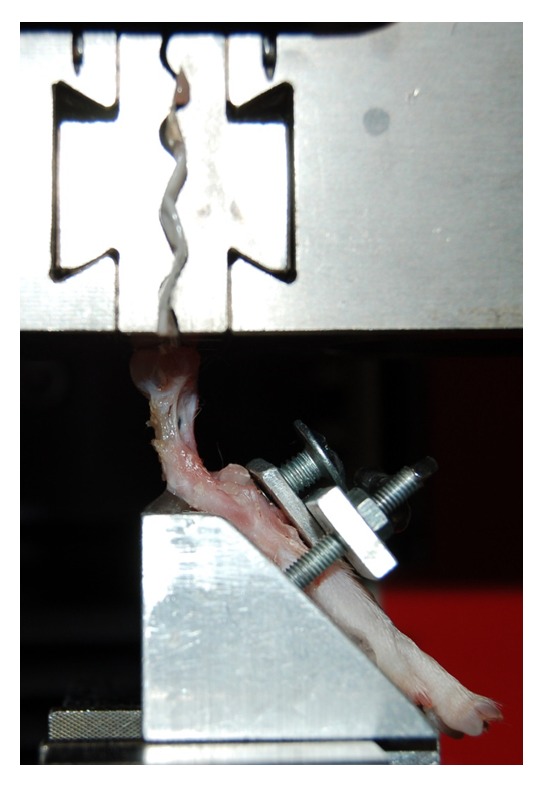
Custom-made setup for tensile testing with mounted Achilles tendon-calcaneus-foot complex.

Prior to testing, the fresh-frozen animal specimens were thawed in a bath of NaCl overnight at 4°C and stored at room temperature for at least 4 hours before preparation. The Achilles tendon-calcaneus-foot complex was dissected from the hind limb. The gastrocnemius-soleus muscle was removed with the blunt end of a scalpel, as described in literature [28]. The proximal end of the tendon was spread out of some paper. The paper was then folded two times and fixed with tape. The foot was mounted with a cyanoacrylate adhesive (LOCTITE® 4902™, Henkel, Düsseldorf, Germany) at 45° to the surface of a custom-made aluminum block and additionally fixed due to a clamping unit via screws. The specimens were mounted on a custom-made test setup in a materials testing machine (Z1.0, Zwick, Ulm, Germany) for tensile testing (Figure 4). All tendons were preloaded with 1 N, and width and thickness were measured with a caliper at three measuring points. Cross-sectional area was calculated from the averaged values under the assumption that the area is oval. Subsequently, the tendons were stretched at a rate of 1 mm/s until complete rupture was observed. Care was taken that the specimens were kept moist with NaCl throughout the procedure. Nonoperated tendons from the bilateral side (left leg) were used as a control (n = 4). Load-displacement curves were recorded and evaluated.

### 2.7. Histological Analysis

The specimens were dehydrated in a graded series of alcohol and embedded in polymethylmethacrylate. Slices in the longitudinal direction of the implant were cut with a laser microtome (TissueSurgeon, LLS ROWIAK GmbH, Hannover, Germany) and stained with Hematoxylin & Eosin (HE). Slice thickness was 10 *μ*m. Scanning and digitalizing for evaluation were performed using a digital microscope (VHX-6000, Keyence, Osaka, Japan) at 500x (objective VH-Z250T) magnification. Samples were evaluated qualitatively in terms of structure and degradation of the scaffold (preserved fiber structure), reaction of surrounding tissue (cell infiltration), and cell migration into scaffolds.

### 2.8. Data Analysis

Statistical analysis was performed using IBM SPSS Statistics 22 software (IBM, Ehningen, Germany). The statistical significance of differences was calculated by Mann–Whitney test within two independent groups. The level of significance was set to p < 0.05.

## 3. Results

### 3.1. Biomechanical Testing of Scaffolds In Vitro

Biomechanical data of the new charges of scaffolds used in this study are displayed in Table 1. The test material shows initial higher mean maximum failure load (Fmax) compared to the control material, but the difference is not significant (p < 0.05). There is also no significant difference of stiffness. Mean tear strength (tensile load normalized to cross section) and elastic modulus of the test material were significantly higher compared to the control material (p < 0.05).

**Figure 5:**
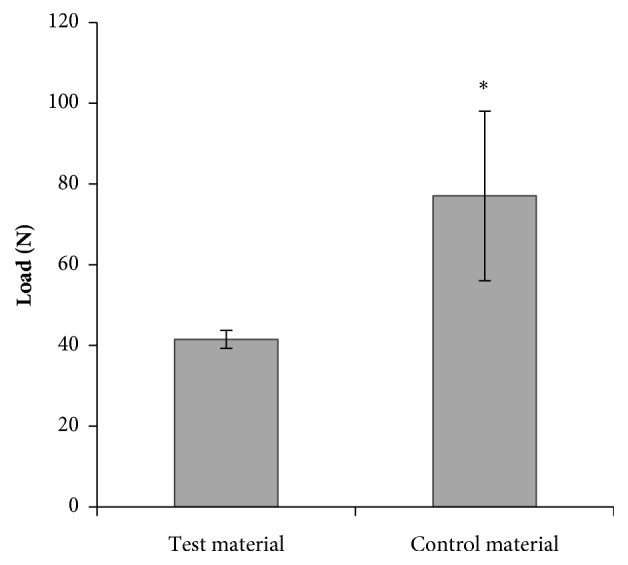
Suture retention strength (maximum failure load, mean ± standard deviation) using a single vertical stitch. *∗* p < 0.05.

The retention strength of single vertical stitches in both scaffold materials (maximum failure load) is demonstrated in Figure 5. The test material showed a lower maximum failure load (41.5 ± 2.2 N) compared to the control material with 77.0 ± 21.0 N. Differences were significant (p < 0.05).

### 3.2. Analysis of MRI Data

**Figure 6:**
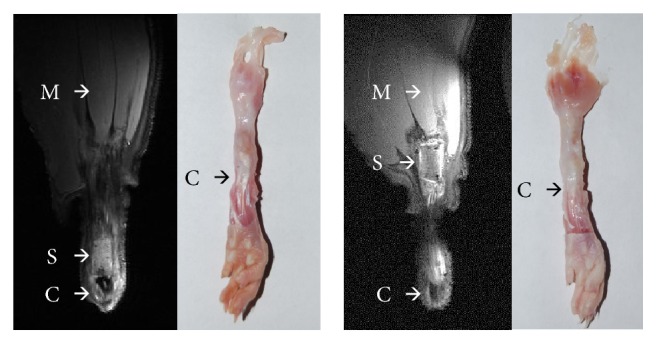
T_2_-weighted MR images and photographs (dorsal view) of experimental hind limbs repaired with collagen scaffolds after 28 days of implantation. Left MRI: scaffold is located distally as implanted and expected. Right MRI: scaffold is located proximally in the gastrocnemius muscle. Both prepared samples (for biomechanical testing) with analogues scaffold position gave no evidence about this position macroscopically. Arrows mark the position of calcaneus (C), scaffold (S), and gastrocnemius muscle (M).

All animals tolerated the surgical procedure. No dropouts or adverse events occurred during the observation period. MR images showed that the location of the scaffolds relative to the tendon insertion on the calcaneus differed considerably in some cases. Some of the scaffolds were located in the proximal part of the lower leg, which was an indication that the reconstruction might have failed distally. Total distances ranged from 3.2 mm to 18 mm (Figure 6).

An overall failure rate of refixation of 48% (13/27) was observed. In one case in the test material group, failure could not be determined due to MR image artifacts. MR analysis indicated that all failures were caused due to suture tear-out. No material defects of scaffolds themselves were visible, independent of the scaffold material. An overview of the location of scaffolds and failures are displayed in Table 2.

### 3.3. AFI

**Figure 7:**
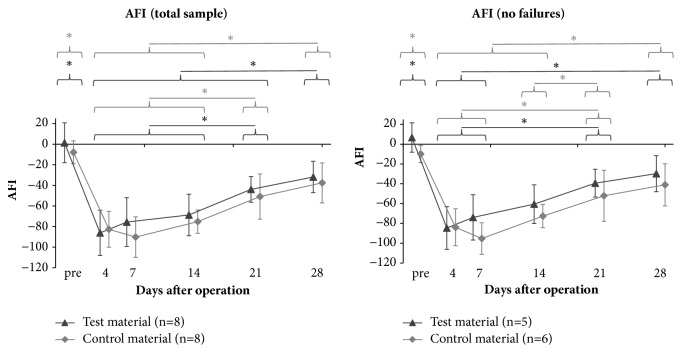
Achilles Functional Index (AFI) at different postoperative observation times after Achilles tendon repair with collagen scaffolds. Symbols represent mean ± standard deviation. *∗* p ≤ 0.05.

Achilles Functional Index values are shown in Figure 7. Differences between the Achilles tendon repairs with the two different scaffold materials were not significant for all time points (p > 0.05).

The results of both groups showed typical curves, as the AFI is neutral preoperatively, decreased significantly (p < 0.01) in the first days after surgery, and recovers over time. The lowest AFI over time was seen on day 4 for the test material group and on day 7 for the control group, respectively. Postoperatively, the increase in AFI of the total sample at two consecutive time points was significant from day 14 to 21 and from day 21 to 28 for the test material (p ≤ 0.05) and from day 14 to 21 for the control material (p < 0.05). The AFI on day 28 was significantly higher compared to all other postoperative days (p ≤ 0.05) for the test material group, and for the control material group AFI on day 28 was higher compared to day 4 up to day 14 (p ≤ 0.01). The AFI on day 28 of both groups was still decreased compared to the preoperative AFI (p < 0.01).

When the failed samples (detected within MRI) were excluded from the analysis retrospectively, both groups still showed a significant difference between preoperative evaluation and day 28 postoperatively, but the difference between the materials was also still not significant (p ≥ 0.126). Only a slight tendency of improved AFI for the test material was found.

### 3.4. Biomechanical Testing

Preparation of samples revealed higher cross section areas of repaired tendons compared to contralateral native tendons (p < 0.01; see Table 3). The surrounding connective tissue could not be distinguished from the scaffold material (Figure 6).

During tensile testing, one sample of the test material group was not correctly mounted in the test setup, so slipping occurred. The sample was excluded from evaluation. In total, healed tendon defects replaced with the test material (n = 7) and the control material (n = 8) showed almost similar maximum tensile loads. The respective native tendons showed only slightly higher tensile loads. Data did not differ significantly between the three groups (p ≥ 0.07). The stiffness of the samples showed no significant differences between the two scaffold materials (p > 0.23). The stiffness of native group was significantly higher (p < 0.01). Tear strength was also significantly reduced in the tendons treated with collagen scaffolds compared to the respective native tendons (p ≤ 0.01). There was no significant difference between the test and control material (p > 0.69). In total, there were only slight differences in the biomechanical data between failed and successful repairs (according to MRI). No significant difference was detected (p > 0.40; see Table 4).

### 3.5. Histology

**Figure 8:**
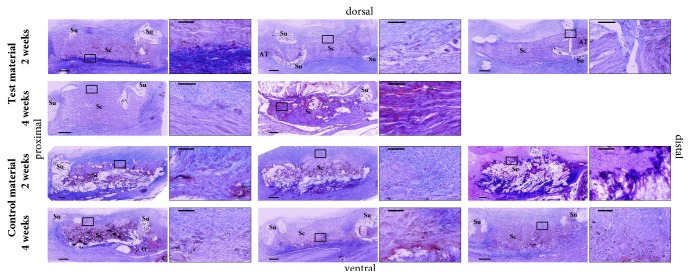
Histological specimens of test material (first two rows) and control material (bottom two rows) obtained two and four weeks after surgery, respectively (HE-staining, original magnification 500x); bar in survey views: 500 *μ*m; bar in detailed views: 100 *μ*m. Sc: scaffold; Su: suture; AT: Achilles tendon; C: calcaneus, O: ossification.

At two weeks postoperatively, the fiber structure of both scaffold materials was clearly visible. Low cell reaction could be observed overall, although the cell reaction on the ventral side was higher compared to the dorsal side. There was only slight to no visible cell migration into both scaffold materials. Furthermore, bridging between the native tendon and scaffold materials with tendon tissues could not be found (Figure 8, first row).

One sample of the test material implanted for four weeks could not be analyzed as the histological preparation failed. One of the remaining two scaffolds was located in the distal part of the lower limb and looked similar compared to the two-week samples. Scaffold structure was well preserved and little cell reaction was observed. Only at the outside margins of the scaffold, some cell migration could be detected. The other sample moved in the proximal part of the limb after failure and showed an obvious rebuilding process. Scaffold fibers were visible at only a few locations. Overall, cell migration into the scaffold was seen (Figure 8, second row).

At two weeks postoperatively, samples of the control material showed a higher surrounding cell reaction, compared to the test material. The scaffolds were in rebuilding process, as there were only few structures visible. Cell migration into the scaffolds was mainly visible at the outer margin (Figure 8, third row).

Within the four-week samples of the control material, there was one failed scaffold located next to the calcaneus. The remodeling process was visible and some ossification occurred on the distal side of the scaffold. The two successful samples showed less remodeling. The fiber structure of the scaffolds was visible and cell migration was seen in deeper scaffold regions (Figure 8, fourth row).

## 4. Discussion

In the present study, a newly introduced scaffold material for tendon augmentation based on bovine cross-linked collagen was tested and compared to a control material based on porcine collagen (DX Reinforcement).

Before animal testing, the scaffold material was tested in vitro, showing promising biomechanical and cell biological properties [25]. The biomechanical data of the test material used in the present study were superior to the DX Reinforcement Matrix material and were in the range of the GraftJacket allograft [8]. In the present study, we used two patches of the control material which differed in their mechanical properties, resulting in high standard deviations in vitro. Also the means of stiffness and tensile modulus were about three times higher compared to the control material [25]. Due to high costs and long delivery times of the commercial control material, the patches were used for both in vitro and in vivo trials to save time and material. The retention strength of single stiches was significantly lower for the used test material compared to the control material. It should be noted that results of the suture retention test [10] are influenced by sample characteristics of the material tested such as thickness; the control material was 1.5 times thicker than the test material. However, in clinical use, single stitch sutures are not commonly used to augment RCR [29]. Therefore, future studies should be carried out with clinically more relevant suture techniques.

MRI allowed a qualitative evaluation of the Achilles tendon repair before the samples were prepared for further testing. In the present study, an overall failure rate of 48% was observed. Most of these failures were not visible during sample preparation (Figure 6), as all animals showed rebuilt tendon and new connective tissue (e.g., scar tissue). By means of MR imaging conducted postmortem at the transected hind limb, elongation of the native tendon was detected, causing dislocation of the scaffolds. The implanted scaffolds themselves remained intact. It was assumed that failures were caused due to suture tear-out. While the evaluation of the distal failure was quite obvious (big distance to calcaneus), the classification of proximal failure was more difficult, because the elongation of the musculotendinous junction was subjected a higher variability. Although the problem of suture failure rates in tendon repair in humans is known [10, 30, 31], this issue is rarely discussed with respect to the outcome of tendon repair in animal models. Therefore, MRI may be a suitable auxiliary tool to validate functional, biomechanical, and histological outcomes. Another enhancement, such as contrast-agent enhanced MRI as described by Cutlip et al. [32], could be suitable for in vivo experiments.

The AFI was shown to be valuable for quantifying the functional performance of the repair over time in the rat model [26]. We used a simple setup using white paper on the floor of the walkway and food dye to color the hind paws. Even after conditioning trials, the rats often stopped and walked backwards to explore the corridor. Therefore, they were sent over the walkway up to three times each time point to obtain at least three left and right printed hind paws for the evaluation. Compared to other studies [28, 33–35], we observed similar functional outcomes with a sharp decrease in AFI in the first postoperative days with improvement over time. In the literature the time to improvement varied from 15 days [28, 33] to 40 days [34], depending on factors like defect size or scaffold material. Return to complete function was nowhere to be found. In our study, AFI also did not achieve initial preoperative values after 28 postoperative days of healing. However, differences in Achilles tendon repair with both different scaffold materials could not be observed. Murrell et al. [26] showed that AFI is sensitive for different groups such as sham-op, repair, and defect. AFI seems to be not sensitive enough to differentiate treatment groups, which differ only in the scaffold material used. Liang et al. [35] therefore introduced a video-based gait analysis with higher sensitivity. Nevertheless, the results coincide with our biomechanical data.

The biomechanical data of the newly developed test material showed similar outcomes compared to the control material. The maximum failure loads of both scaffold materials were in the range of native tendon. This is in agreement with the results of Best el al. [28] who investigated a simple repair of a division of the Achilles tendon in rats. However, in a study by Webb et al. [34], the maximum failure loads of the repaired tendons at 40 days postoperatively using several synthetic scaffolds in an Achilles tendon defect model were significantly decreased compared to the native control.

In our study, the tear strength and stiffness of native tendons were significantly higher compared to the repaired tendons. In this context, our animal study was limited by missing a negative control group. Aspenberg and Virchenko [36] showed in their investigation that a 3 mm defect without repair achieved 70% of the force at failure of unoperated tendons after 28 days postoperatively. For further investigations, negative control groups (i.e., tendon repair without a scaffold) should be attempted to determine the biomechanical properties of the native scar and connective tissue.

Our histological findings are limited due to a small sample size and high failure rates. We assume that cell infiltration, remodeling, and tissue organization of newly formed ECM are highly dependent on whether the Achilles tendon repair using scaffolds was intact over time, particularly at the junction with native tendon tissue, and whether it transferred tensile load. The histological evaluation of the in vivo host response to several collagen scaffold materials was performed in a defect in the musculotendinous tissue of the abdominal wall by Valentin et al. [37], but this model lacked tensile and strain loads applied to the grafts, like in tendons [38]. Although our results with respect to the remodeling and degradation process were inconsistent in some cases, the test material seemed to undergo a slower remodeling process, which was expected due to the processing of the test material by means of cross-linking. ECM material that is further processed to minimize its degradation rate, e.g., through cross-linking, is associated with fibrous encapsulation and chronic inflammation [39]. Therefore, removal of potential free epoxide was carried out by successive washing. However, neither the test material nor the control material showed any signs of inflammation. For further histological investigations not only a higher sample size, but also a preparation with additional staining (e.g., Picrosirius red) is recommended, which allows a more detailed analysis, like quantitative analysis of cell migration and evaluation of new versus old collagen.

The relatively high implant failure rate observed in our study was caused by the limitations of the animal model. The scaffolds were applied as an interposition in a large tendon defect. For tendon repair of the Achilles tendon in rats, defect sizes range from 3 mm [36] to 5 mm [34]. The defect size of 5 mm was considered suitable as we used comparatively big rats (mean weight operation about 400 g). Thus, healthy tissue, which is important for secure anchoring of the sutures, was resected. Furthermore, the M. plantaris tendon was removed to prevent any negative impact as splints [26]. As the space in small animals is limited, we used only a simple stitch suture technique as described in [34, 40]. Suture techniques using more stiches preventing suture tear-out are recommended [41]. Our animals were allowed to move free in their cages postoperatively. Some animal models examine if postoperative immobilization may improve the outcome of tendon regeneration [38, 42], but in rodent studies, immobilization due to several casting methods resulted in skin irritation, weight loss, slipping out of the cast, or muscle trophy due to the resting position of the limbs [43]. In addition, we used anesthesia that could be directly antagonized after the surgical procedure. Thus, the animals spent less time under anesthesia after the operation, which incurred fewer perioperative risks like hypothermia. Since the animals were supplied with analgesics, this allowed them to stress their operated leg immediately postoperatively and may have promoted rupture of the sutures. In a subsequent investigation we tested the primary fixation stability of different suture techniques for the described animal model [44]. The results support the findings that almost all samples failed due to suture tear-out of the tendon and simple sutures performed poorly against techniques with more suture strands. Therefore, it is important to use a secure suture technique to decrease the risk and prevent suture tear-out or other defects in vivo. In our defect model we did not consider the normal anatomy of the native Achilles tendon of the rat, which consist of three subtendons. It was reported that they cause nonuniform behavior (relative displacement and differential strains) within the tendon [45]. Further investigation may include influence of the subtendon organization and properties on tendon defect models. For further investigations, large animal models should also be considered to assess the application of scaffolds for tendon repair in a situation closer to that of humans.

## 5. Conclusion

We analyzed a newly introduced bovine collagen scaffold material for tendon repair in an Achilles tendon defect model in rats. The experimental data revealed that the bovine scaffold material had comparable biomechanical and biological properties in vitro and in vivo compared to a commercially available porcine scaffold material. In order to detect possible failure of the replaced tendon and therefore to validate the functional, biomechanical, and histological outcomes, MRI as an auxiliary measurement tool is recommended. Further in vivo investigations should be carried out to assess the degradation and remodeling process of the scaffolds in detail.

## Figures and Tables

**Table 1:** Biomechanical data (mean ± standard deviation) of the two collagen scaffold materials. Fmax: maximal failure load, Rm: tear strength, S: stiffness, EM: elastic modulus. *∗* p < 0.05.

**Scaffold**	**Fmax (N)**	**Rm (N/mm** ^**2**^ **)**	**S (N/mm)**	**EM (MPa)**
**Test Material**	395.6 ± 70.5	22.7 ± 4.9*∗*	75.0 ± 10.1	90.4 ± 12.1*∗*
**Control Material**	309.8 ± 193.9	10.4 ± 5.5	79.4 ± 30.6	61.7 ± 13.5

**Table 2:** MRI analysis: location of the scaffolds and failure rates of the samples (n.d.: nondescript).

**Material**	**position in vivo**	**n**	**n (failure)**	**n (no failure)**	**n (n.d.)**
**Test Material**	proximal	6	5	-	1
	distal	8	2	6	-

**Control Material**	proximal	3	3	-	-
	distal	11	3	8	-

**Table 3:** Cross section area (mean ± standard deviation) of explanted Achilles tendons treated with both collagen scaffold materials compared to contralateral native Achilles tendon. *∗∗* p < 0.01.

	**Test Material**	**Control Material**	**Native Tendon**
**Cross section (mm** ^**2**^ **)**	22.93 ± 4.71	22.75 ± 4.69	6.76 ± 3.01*∗∗*

**Table 4:** Biomechanical data (mean ± standard deviation) of the Achilles tendon defects treated with collagen scaffolds after 28 days postoperatively compared to native tendons. Sample sizes: native) n = 4; Test Material) total: n = 7, not failed: n = 4, failed: n = 3; Control Material) total: n = 8, not failed: n = 6, failed: n = 2. *∗∗* p < 0.01.

	**Test Material**	**Control Material **	**Native Tendon**
**Failure load (N)**			
total	54.5 ± 16.4	63.1 ± 19.5	76.6 ± 11.6
not failed	55,7 ± 18.7	67.7 ± 20.2	
failed	52.9 ± 16.7	49.2 ± 10.3	

**Stiffness (N/mm)**			
total	9.0 ± 2.8	10.7 ± 2.7	20.2 ± 6.6*∗∗*
not failed	8.6 ± 1.8	11.1 ± 2.5	
failed	9.6 ± 4.3	9.4 ± 4.0	

**Tear strength (N/mm** ^**2**^ **)**			
total	2.5 ± 0.8	2.8 ± 1.0	13.3 ± 5.9*∗∗*
not failed	2.2 ± 0.9	2.9 ± 1.2	
failed	2.8 ± 06	2.6 ± 0.5	

## Data Availability

The data used to support the findings of this study are included within the article.
